# Surgical Treatment Options for Subacute Ischemia of the Hand: Case Report and Literature Review

**Published:** 2010-04-12

**Authors:** Ashley A. Dunn, Kyle A. Belek, Zlatko Devcic, Samira Rathnayake, Jennifer H. Kuo, Mauricio Kuri, David S. Chang, Scott L. Hansen

**Affiliations:** Department of Surgery, University of California San Francisco

## Abstract

**Purpose:** The most effective surgical approach to the treatment of digital ischemia has not yet been established. The purpose of this study is to review currently accepted options for revascularization in acute and chronic settings of digital ischemia, and to augment this discussion by describing the approach of our surgical team in a unique case of subacute ischemia. **Operative Technique:** To restore blood flow to a patient's ischemic hand, we performed a microvascular reconstruction, using a reverse interpositional vein graft with 3 anastomoses: the ulnar artery was used for inflow and the superficial palmar arch and the common digital artery were used for outflow. **Results:** The patient experienced immediate postoperative pain relief. Blood flow was restored, which prevented digital amputation. The graft remained patent at 18 months' follow-up and the patient exhibited normal motor and sensory function. **Conclusions:** Surgical reconstruction of the hand is a viable treatment option for carefully selected patients presenting with subacute digital ischemia. Other medical and surgical techniques have been described in the recent literature, but further study is needed to determine the long-term success of newer microsurgical interventions.

Digital ischemia of the hand has a number of causes, including vascular disease, thromboembolism, acute infection, and trauma. Patients often present with finger pain, discoloration, numbness, tingling, and, in advanced cases, ulceration and necrosis. If left untreated, severe cases of digital ischemia can lead to tissue loss or amputation.^1^ In comparison to lower extremity ischemia, upper extremity ischemia, including ischemia of the digits, is relatively rare and has received much less attention in the vascular literature.^2^,^3^ The purpose of this article is to describe the options for revascularization in acute and chronic settings of hand ischemia. We will use a unique case of subacute digital ischemia to illustrate one approach to this condition.

## CASE REPORT

**Figure 1:**
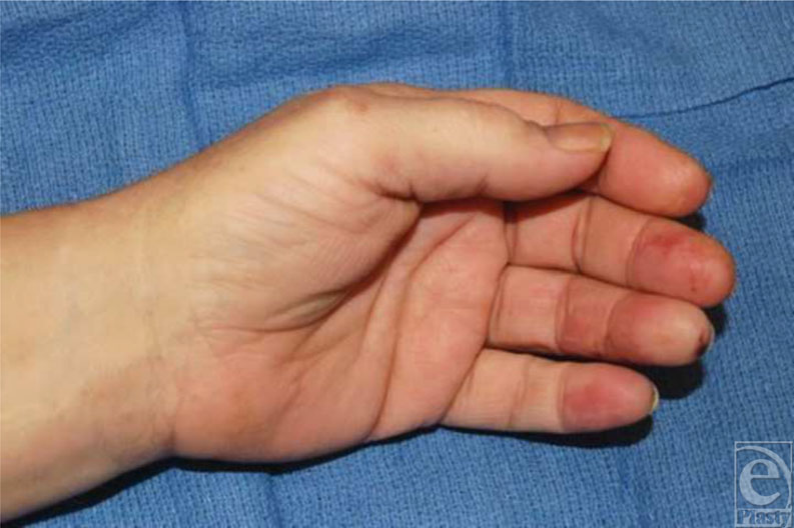
Preoperative photograph showing cyanosis and ulcerations in the fingertips resulting from ischemia.

**Figure 2:**
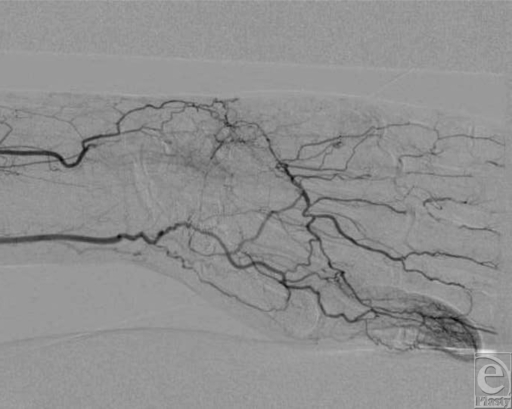
Left hand angiogram demonstrating occlusion of the radial and ulnar arteries at the wrist.

A 66-year-old, right-hand-dominant man presented with dull, throbbing pain in his middle, ring, and small fingers of his left hand, which began 2 days prior to admission. On physical examination, the affected digits were cyanotic with ulceration (Fig 1). Radial and ulnar pulses were palpable. The patient's medical history was significant for smoking, coronary artery disease, and hypertension. He also reported sustaining a deep laceration of the ulnar aspect of the left wrist with a piece of glass at age 10 years. Arteriography of his upper left extremity demonstrated occlusion of the ulnar and radial arteries at the level of the wrist, with poor distal perfusion to the middle, ring, and small fingers (Fig 2). Distal collateralization beyond the level of occlusion at the wrist suggested chronic ulnar artery occlusion. There was no filling of the superficial palmar arch. The patient had a normal electrocardiogram. Both the aortogram and echocardiogram showed no evidence of an embolic source. We surmised that the patient's abnormal arterial anatomy was caused by a previous ulnar artery injury with subsequent atherosclerotic disease leading to subacute digital ischemia. Given these findings, the surgical team recommended an attempt at distal revascularization of the digits to prevent further tissue loss.

An ulnar artery to superficial palmar arch bypass using a cephalic vein graft from the left forearm was performed. Initial dissection was carried out under tourniquet control with release prior to anastomosis. Access to the superficial arch was obtained through a palmar incision, incorporating the carpal tunnel. There were several thrombi in the ulnar artery, including one just proximal to the superficial arch and another in the distal wrist. The ulnar artery appeared to be thrombosed in the distal forearm and proximal wrist and up into the proximal hand. However, the proximal ulnar artery had pulsations and a strong Doppler signal. The distal superficial palmar arch also had a good Doppler signal, suggesting a patent artery, likely from collaterals. The distal ulnar artery was therefore used for inflow and the proximal superficial arch for outflow.

**Figure 3:**
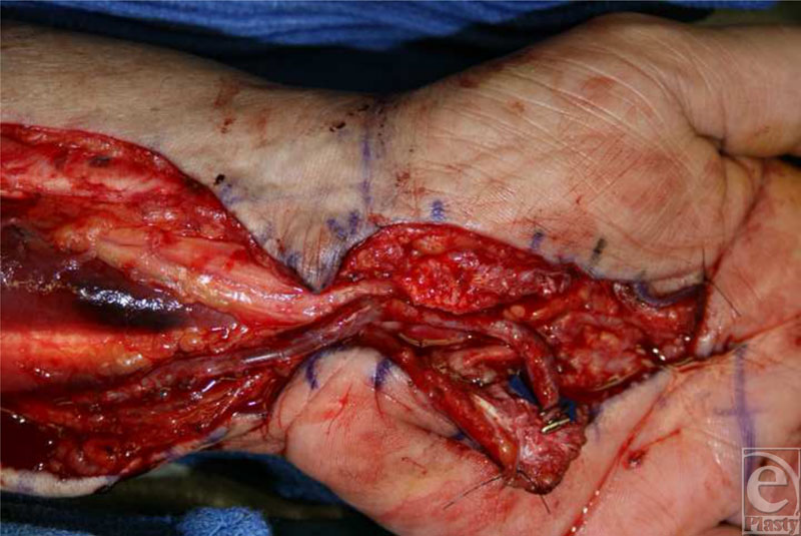
Intraoperative photograph illustrating microsurgical bypass of the ulnar artery to the superficial palmar arch using a cephalic vein graft.

A segment of cephalic vein was harvested from the left forearm. The ulnar artery was divided just proximal to the area of thrombosis and the superficial arch just distal to the thrombosis. These vessels exhibited pulsatile bleeding and both had grossly normal intima. The vein graft was reversed and anastomosed in an end-to-end fashion to the distal ulnar artery, using interrupted 9-0 nylon sutures with the operating microscope. The vein graft was then brought down through the carpal tunnel into the palm (Fig 3). Because of a size mismatch, the palmar arch was anastomosed to a side branch on the cephalic vein graft in an end-to-end fashion. It was unknown how much perfusion would reach the small finger so the remaining distal portion of the cephalic vein was used to create a third anastomosis to the common digital artery to the ring and small fingers. Because of the size mismatch, the common digital artery was divided and anastomosed to the side of the cephalic vein graft. The distal end of the graft was then ligated. A handheld Doppler device confirmed pulsatile flow through the graft and distal to the anastomoses. Immediately, capillary refill to the ring and small fingers was brisk. An implantable Doppler probe was placed on the vein graft to monitor graft patency postoperatively.

**Figure 4:**
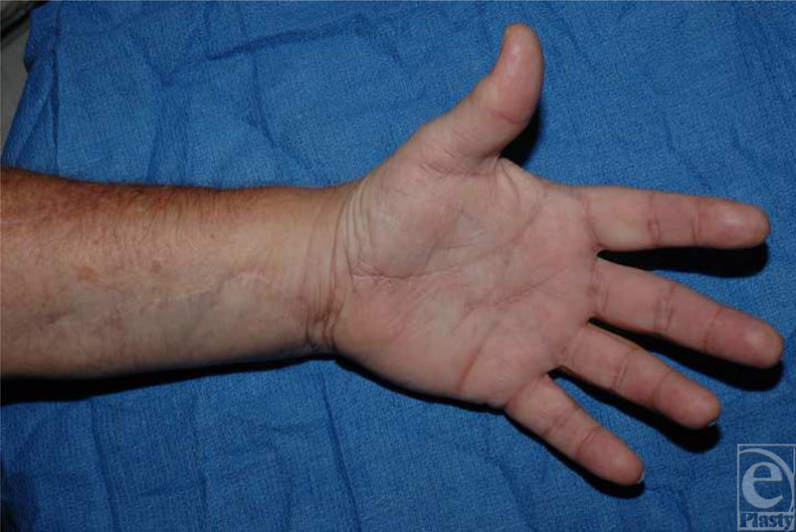
Eighteen months' follow-up reveals patent graft with adequate perfusion and full motor and neurological function.

The patient has since recovered uneventfully and without complication. The graft has remained patent at 18 months' follow-up and continues to have brisk capillary refill to the involved digits. His ulcerations have healed (Fig 4). The finding from the patient's neurovascular and motor examination is normal.

## DISCUSSION

The term *subacute ischemia* generally describes a clinical situation in which chronic ischemia is exacerbated by 1 or more factors, such as atherosclerosis, which then leads to acute ischemia. This is distinct from chronic ischemia, in which mild symptoms develop over a long period of time such that there is no immediate danger of tissue loss, and acute ischemia, in which tissue loss will occur if blood flow is not restored within hours. In our case report, the patient had a chronic occlusion of his ulnar artery secondary to trauma. Progression of atherosclerosis in his hand eventually led to critical ischemia and tissue loss.

The initial treatment for digital ischemia depends on the etiology. Patients presenting with exaggerated digital vasospasm (as in Raynaud's phenomenon) can be treated with calcium channel blockers or, more recently, with botulinum toxin type A (BTX-A).^4^ Other medical management has traditionally involved β-blockers, antiplatelet therapy, thermal biofeedback, and avoiding tobacco and alcohol consumption. Acute embolism is treated with thrombectomy. Additional management of these patients includes management of the underlying cause. Emboli from the heart can arise from arrhythmia or mechanical valves, and these can be treated with systemic anticoagulants. Atheroembolism can also be treated with intravascular stents.

Arterial reconstruction should be considered for patients with ischemia and tissue loss that signals that digital amputation is imminent if increased perfusion is not achieved.^5^,^6^ Microsurgical reconstruction for the upper limbs has historically received little attention despite the numerous similarities that exist between upper and lower extremity ischemia.^2^,^7^ Only a few have attempted to explore the subject, starting with Garret et al^8^ in 1965.^6^,^9^ Prior to the adaptation of cardiac bypass grafting techniques for peripheral revascularization, the standard protocol for treating digital ischemia was simple resection of the occluded segment.^6^ The technique is still used to treat radial or ulnar artery aneurysms, but problems related to vessel tension across the wrist make this technique undesirable in patients with large areas of occlusion requiring excision.^10^

In 1989, Jones and Emerson^6^ described 14 chronically ischemic patients who underwent distal radial or ulnar artery revascularization. He found immediate postoperative improvement of symptoms in a majority of patients, although 5 grafts (35%) eventually thrombosed. Other more recent studies of reverse interpositional grafts involving resection of blocked arterial segments note long-term patency rates of 48%–100% at various follow-up intervals.^11^^-^14 The long-term success of these grafts depends on a number of factors. Dethmers and Houpt^15^ have documented a correlation between longer grafts and long-term occlusion. Similarly, Hughes et al^2^ found that patients revascularized for upper extremity ischemia caused by trauma had better outcomes than patients treated for atherosclerotic disease.

Recently, several examples of microsurgical bypass grafts that do not include excision of the diseased segment have been described.^5^,^9^,^10^ The reported benefits of leaving the occluded segment intact include an accompanying decrease in operative time and the ability to revascularize the hand without disrupting collateral flow. Katz and Kohl^16^ documented the outcomes of 32 patients treated for acute hand ischemia, reporting successful digital reperfusion in all patients and 100% graft patency at follow-ups from 6 months to 8 years. Despite the advantages of the bypass graft, however, aneurysmal arteries at the wrist may require resection because of the risk of embolization to the digital arteries.

The choice of graft ultimately depends on the availability of autologous conduit and the size of the vessel being bypassed. The saphenous and cephalic veins are currently the most frequently used autologous conduits for upper extremity bypass operations.^1^,^3^ Venous conduits are not limited by length, and saphenous veins generally have a more compatible diameter than cephalic veins.^5^ Where vessel length is not an issue, arteries can be ideal for interpositional or bypass grafting because they are structurally homologous to the affected vessels. Smith and colleagues^17^ found no evidence of intimal hyperplasia and 100% graft patency from 7 to 24 months in 3 patients with hypothenar hammer syndrome treated with a segment of the deep inferior epigastric artery. These conduits could thus be indicated in patients who may need additional procedures in the future, such as smokers or manual laborers who must continue to expose the hand to repetitive palmar trauma.

Given the relatively recent history of microsurgical reconstruction for digital ischemia, it will take time to assess the long-term success of interpositional and bypass grafts. Specifically, it will be important to determine the longevity of various types of grafts distal to the wrist, since the hand is particularly prone to trauma. Superficial grafts that cross the wrist will also need to be scrutinized for occlusion caused by flexion and extension. As expected, treatment options should be considered on the basis of the etiology and severity of the patient's ischemia. In carefully selected patients with subacute ischemia, microsurgical revascularization should be considered to prevent tissue loss.
